# Identification of sources of lead exposure in French children by lead isotope analysis: a cross-sectional study

**DOI:** 10.1186/1476-069X-10-75

**Published:** 2011-08-28

**Authors:** Youssef Oulhote, Barbara Le Bot, Joel Poupon, Jean-Paul Lucas, Corinne Mandin, Anne Etchevers, Denis Zmirou-Navier, Philippe Glorennec

**Affiliations:** 1 EHESP - School of Public Health, Sorbonne Paris Cité, 35043 Rennes, France; 2 IRSET- Environmental and Occupational Health Research Institute, 35000, Rennes, France; 3 INSERM- French National Institute of Health and Medical Research, U954 Nancy University Medical School, 54505 Vandoeuvre Les Nancy, France; 4 Lariboisière Hospital (AP-HP), Toxicology laboratory, 75475 Paris, France; 5 Paris Est University - CSTB - Scientific and Technical Building Centre, 77447 Marne-la-Vallée, France; 6 EA 4275 Biostatistics, Clinical Research and Subjective Measures in Health, Nantes University, 44035 Nantes, France; 7 InVS - French Institute for Public Health Surveillance, 94415 Saint Maurice, France; 8 Nancy University Medical School, 54505 Vandoeuvre Les Nancy, France; 9 INSERM- French National Institute of Health and Medical Research, U625 (GERHM), Rennes 1 University, 35042 Rennes, France

## Abstract

**Background:**

The amount of lead in the environment has decreased significantly in recent years, and so did exposure. However, there is no known safe exposure level and, therefore, the exposure of children to lead, although low, remains a major public health issue. With the lower levels of exposure, it is becoming more difficult to identify lead sources and new approaches may be required for preventive action. This study assessed the usefulness of lead isotope ratios for identifying sources of lead using data from a nationwide sample of French children aged from six months to six years with blood lead levels ≥25 μg/L.

**Methods:**

Blood samples were taken from 125 children, representing about 600,000 French children; environmental samples were taken from their homes and personal information was collected. Lead isotope ratios were determined using quadrupole ICP-MS (inductively coupled plasma - mass spectrometry) and the isotopic signatures of potential sources of exposure were matched with those of blood in order to identify the most likely sources.

**Results:**

In addition to the interpretation of lead concentrations, lead isotope ratios were potentially of use for 57% of children aged from six months to six years with blood lead level ≥ 25 μg/L (7% of overall children in France, about 332,000 children), with at least one potential source of lead and sufficiently well discriminated lead isotope ratios. Lead isotope ratios revealed a single suspected source of exposure for 32% of the subjects and were able to eliminate at least one unlikely source of exposure for 30% of the children.

**Conclusions:**

In France, lead isotope ratios could provide valuable additional information in about a third of routine environmental investigations.

## Background

Lead is a ubiquitous versatile heavy metal. It has been widely used since 3500 BC [1]. It is the most studied environmental pollutant and its adverse health effects are well documented [2]. High exposure to lead damages almost all organs and organ systems, especially the central nervous system, kidneys and blood cells [3]. Despite considerable reduction of the amount of lead in the environment as a result of control measures and policies (in particular, ban on lead-based paints and phase-out of leaded petrol), environmental lead exposure remains an important public health issue.

It is well established that there is no known safe exposure to lead [4,5]. Several studies have shown effects at very low doses, even below the established blood lead level (B-Pb) limit for action of 100 μg/L (0.48 μmol/L); this intervention level should not be considered as a threshold for the harmful effects of lead [6]. These effects concern cognitive and neurobehavioral deficits, lower intelligence quotient scores, fine motor skills and a wide range of other [7-11]. Children are more vulnerable because of their greater contact with their environment (hand-mouth behavior), their higher intake rate and the development of their neural system. Recently, the European food safety agency (EFSA) established a benchmark dose: an increase of 12 μg/L could decrease the IQ score by one unit, without threshold below which neurodevelopmental toxicity could be defended [12].

In some countries, there are many complex sources of lead exposure (mining activities, pollution from leaded gasoline remaining in the atmosphere and industrial emissions, cosmetics, etc). In addition, lead-based paint is considered to be the primary lead source for children with B-Pb≥100 μg/L in France and the USA [13] in non industrial environments.

In France, a national survey involving 3,800 children was set up in 2007 by the French Institute for Public Health Surveillance (InVS) to evaluate the prevalence of lead poisoning in children. An environmental survey coordinated by the French Building Research Centre (CSTB) was conducted in about 500 homes of children taking part in the national survey in order to determine the main determinants of current B-Pb. This national survey estimated the geometric mean for the B-Pb of children in France at 15 μg/L, and the prevalence of high B-Pb (≥100 μg/L) was 0.11% [14]. An update of current knowledge of the determinants of these low B-Pb among French children is essential. Furthermore, moderate B-Pb (< 100 μg/L) are becoming a growing public health concern because there is no known safe exposure level. There is, therefore, considerable interest in the development of new approaches to identify sources of lower doses of lead. Routine identification of sources of lead exposure in France is currently based on children's behavior, observation of their homes and determination of lead concentrations in their environment, such as paint, dust, soil, and water. New techniques could be used in addition to these standard approaches to identify sources of exposure in the case of low B-Pb, particularly as environmental health services in some European countries may consider B-Pb below the current limit of 100 μg/L in the course of their screening activities [15].

Lead isotope ratios (LIR) could be a useful means of identifying sources of exposure for individual cases in routine investigations [16]. Numerous studies have demonstrated the usefulness of isotopic signatures for identifying lead exposure sources in mining regions and in homes [17-21]. However, it is more difficult to assess sources of low B-Pb within a restricted area such as the child's home: this approach is more likely to be successful when the potential sources are few and isotopically distinct [16]. In addition, several studies showed that use of LIR technique could be indecisive for B-Pb below 50 μg/L, and that lead isotopic profiles in blood could be easily perturbed by relatively small changes of environmental exposure [22,23].

Actually, LIR could reveal the exposure sources but there is an uncertainty about the effectiveness of this method for prevention purposes at current B-Pb.

The purpose of this study is to assess the proportion of cases where LIR could bring additional insights on exposure sources, with results representative of French children population (aged from six months six years) with moderated B-Pb (25-100 μg/L). This study assessed the usefulness of LIR measurements for prevention in routine environmental investigations, with relatively low analytical cost, so widespread ICP/MS analytical technique was used. More generally, it also aims at improving knowledge of predominant exposure media for children with moderate B-Pb.

## Material & methods

### Population

Children with B-Pb ≥25 μg/L (0.12 μmol/L) were sampled from the children enrolled in the InVS national survey (B-Pb geometric mean for the whole population: 15 μg/L). A two-stage sampling, stratified by hospital and French administrative regions, was conducted for this survey. Hospitals located in areas with a higher risk of lead exposure in housing were intentionally over-represented; the inclusion and survey procedures are described by Etchevers et al. [14]. The sub-population for the complete, validated environmental investigation included 484 children between six months and six years old. One hundred and twenty five (125) of these were included in this study because their B-Pb were above 25 μg/L, representing 12% of French children in this age group according to the survey design (cf. Statistical analyses below).

The parents of children who took part in the study were informed about the purposes of the study and gave their consent. An individual written report on the results was sent to each family. Authorization from the Commission Nationale de l'Informatique et des Libertés (CNIL - French Freedom of Information Commission) was also obtained.

### Environmental sampling

The first step of the environmental survey was to interview one adult living with the child. The questionnaire included information about the child, his/her behavioral habits, family history, and educational level of parents and description of the home. The second step was to inspect the premises to identify the presence of lead in the walls, floors, etc. In each home, up to five rooms were selected using the US-HUD protocol [24] in the following order: child's bedroom, living room, hall, kitchen and bedroom of the brother/sister immediately younger or older. Finally measurements were taken and samples were collected:

- One wiped dust sample of the floor where the child played,

- X-ray fluorescence (XRF) measurements [25] for all painted surfaces and samples of damaged paint that were over 1 mg/cm^2^.

If the child lived in an apartment, a dust sample was collected in the stairwell and XRF measurements were also performed. If one or more balconies of the home had layers of lead-based paint and XRF measurements were positive, an additional flake of paint was collected if possible, with the permission of the occupant. If the child played outdoors in a garden or playground in the close vicinity of the home, the ground was sampled using a ring (2 cm deep) or wipe (0.1 m^2^) for hard surfaces. A sample of the tap water was systematically collected: after 30 minutes without using any water, 2 L were drawntaken, homogenized in a 2 L flask and then poured into a 0.25 L acidified flask. Finally, where appropriate, cosmetics (kohl) or traditional dishes known to be potential sources of lead were also collected.

The concentrations in leachable lead (digestion method described by Le Bot et al. [26]) in samples (excluding tap water for which total lead was measured) collected in the environment of children with B-Pb ≥ 25 μg/L (n = 125) are described in Table 1.

**Table 1:** Distribution of leachable lead concentration in the various types of source for children with B-Pb ≥ 25 μg/L.

			Quantile							
**Type of source (n)**	**Unit**	**LOQ**	**Min**	**25%**	**50%**	**75%**	**90%**	**Max**	**Mean**	**Geometric Mean**

**Tap water (124)**	**μg/L**	1	0.5	0.5	0.5	2.75	7.5	74	4.1	1.3 (1.03-1.6)

**Home dust (469)**	**μg/m^2^**	1	0.5	5	11	26	66	3204	42.7	12 (10.7-13.6)

**Dust from communal** **areas (57)**	**μg/m^2^**	1	4	13	26	64.5	306	1103	94.3	33.7 (24.1-47.1)

**Outdoor soil (81)**	**mg/kg**	0.5	2.1	13.9	29	81.6	169	395	66.2	33.9 (26.2-43.8)

**Wipe of outdoor ground (13)**	**μg/m^2^**	1	7	26.5	115	181	2132	3172	361.5	87.6 (31.6-243)

**House paint (27)**	**mg/g**	0.05	0.03	2.5	14	54.2	102	149	33.1	9.84 (4.2-22.9)

France, 2008-2009.

a) Data not shown for unusual sources

b) Concentrations for samples below the limit of quantification (LOQ) were replaced by LOQ/2.

Isotopic analysis of an environmental sample is relevant only if the lead concentration can lead to B-Pb exceeding or equal to 25 μg/L. We calculated, following the method described by Glorennec et al. [15], concentrations of concern with a Physiologically Based Pharmacokinetic Model (IEUBK-version win 1_1build11 [27]): 4 μg/L for water, 40 μg/m^2 ^for dust, 25 μg/g for soil and 1 mg/cm^2 ^for paints. LIR were then measured for environmental samples whose concentrations were greater than these concentrations of concern, except for samples from unusual sources of poisoning (cosmetics, traditional dishes, etc), that were all analyzed.

### Analytical techniques

#### Blood

At least 1 ml of whole blood was collected in a tube with anticoagulant (EDTA). The isotope ratios were determined using quadrupole ICP-MS (Inductively Coupled Plasma - Mass Spectrometry) (Elan DRCe, Perkin Elmer^®^). The mass bias was corrected with a certified reference material (Common Lead Isotopic Standard, SRM 981, NIST) using the standard bracketing technique described in [28]. Details of procedures of digestion, experimental conditions and accuracy assessment were described in a technical report [29]. Relative standard deviations (RSD) for LIR in blood were respectively between 0.1% and 0.6% for LIR without ^204^Pb and between 0.2% and 0.9% for LIR including the isotope ^204^Pb.

### Environmental samples

Measurement of the leachable lead isotope ratios in the sample digests was performed using quadrupole ICP-MS (Agilent Technology 7500ce). Intercalibrated LIR measurements were performed by the two laboratories assaying lead in the blood and in environmental samples. Intercalibration was conducted in a blood sample after digestion and on an aqueous sample. Each of the two laboratories used its own method for determining mass correction with the standard SRM 981 and correction of blanks. The results were comparable (details are provided in additional file 1 and technical report [29]). Relative standard deviations (RSD) of LIR in environmental samples were respectively between 0.1% and 0.5% for LIR without ^204^Pb and between 0.1% and 0.9% for LIR including the isotope ^204^Pb.

### Interpretation of LIRs for each child

**Figure 1:**
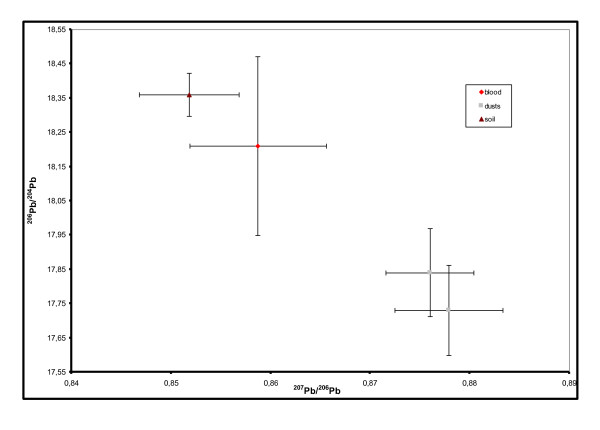
**Graphical plot of ^207^Pb/^206^Pb Vs. ^206^Pb/^204^Pb for a child (example)**. *Error bars represent error measurement (2SD). The soil sample was incriminated as the source of exposure, while the two dust samples from child's house rooms were discarded*.

To identify sources of lead exposure, the isotopic signature of the child's blood was compared with the environmental samples collected in the home. The compatibility between blood and potential sources of exposure (with lead concentrations higher than concentrations of concern) was assessed by comparing the isotope ratios of the four lead isotopes with 95% confidence intervals established using the analytical uncertainty (Ua = two Standard Deviations of measurement of a triple replicate [28]) to determine whether there was an "overlap" between the confidence intervals of the LIRs of the blood and potential sources (see Figure 1). A source was considered compatible, and therefore a suspected source, when its LIR confidence interval overlapped the blood LIR confidence interval.

The most discriminating LIR for each home was selected by calculating a discriminating factor (DF) for each child's home and each LIR, This DF aims at establishing whether the LIR method could be applied or not, by comparing the magnitude of variability of isotopic signatures between sources from the home and the analytical uncertainty of isotope ratios measurement. It is defined as:

(1) DF=Vs∕Ua

where

Vs is the intra-home variability between samples which is defined as the Coefficient of Variation of LIR of environmental samples (water, dust, soil...) from a child's home. It illustrates the variability of LIR between sources from this home.

Ua is the analytical uncertainty (mean of relative standard deviations of LIR calculated on 3 replicates [28,29] of each collected environmental samples from the child's home).

**Figure 2:**
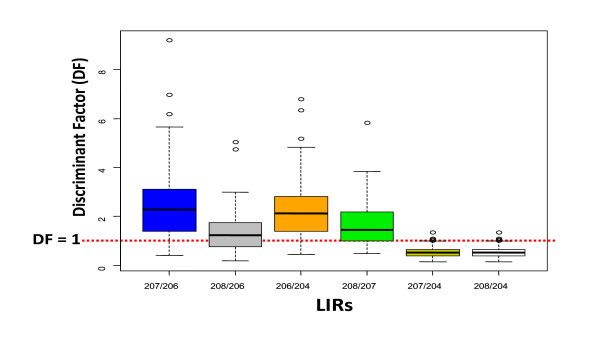
**Discriminating factors of Lead Isotope Ratios. France, 2008-2009**.

When DF≤1, the LIRs were considered unable to identify the sources of exposure because the variability of the isotopic signatures between sources was of the same order of magnitude as analytical uncertainties. The distribution of discriminating factors for all LIRs is given in Figure 2, showing that ^207^Pb/^206^Pb and ^206^Pb/^204^Pb were the most discriminating LIRs.

The use of LIRs was considered "useful" when it permitted to eliminate at least one potential source (whose concentration was greater than the established threshold concentration). Actually, eliminating a source is as important as identifying one since it avoids unnecessary, and possibly harmful removal work to be undertaken in the dwelling. The use of concentrations and LIR was considered "sufficient" when a single source of exposure was probably identified, i.e. only one potential source has concentration greater than concentration of concern and LIR compatible with those from blood. When dust and paint from the same room were both isotopically compatible with the blood, the source was considered to be identified (as a paint, single source). The same applied to outdoor ground and home dust (ground outdoors, single source).

### Statistical analyses

The child by child data analyses and interpretation were based on graphical plots (eg Figure 1) using Excel^®^. These individual results were used to calculate in the population of French children between six months and six years: i) the proportion of children for whom the LIRs were useful; ii) the proportion of children for whom concentrations and LIRs were sufficient. Components of the sampling design (sampling weights, stratification and stages) were taken into account with the "survey" package of R^® ^2.9.0 software [30] to calculate the proportions and their variances.

## Results

### Data description

**Figure 3:**
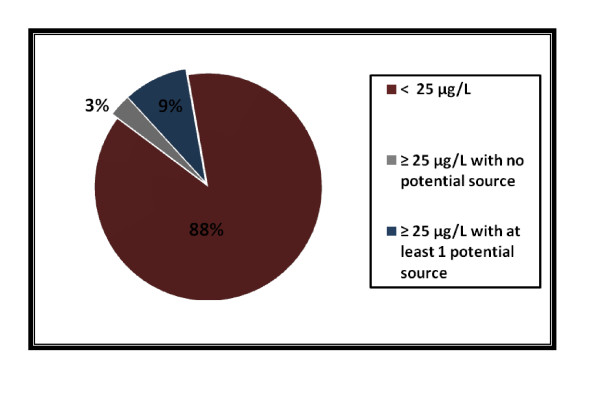
**Children's B-Pb and potential lead sources (n = 484, N = 4,923,058), France, 2008-2009**.

Among the 484 children enrolled in the survey, 125 had B-Pb ≥ 25 μg/L, (with an estimated geometric mean of 35 μg/L in the population). Twenty five percent of the 125 children had no identified potential source (all the collected environmental samples had lead concentrations below concentrations of concern) in their home (Figure 3). Of the 125 homes, 87 were single dwellings and 36 were apartments in collective buildings (2 were not defined).

**Figure 4:**
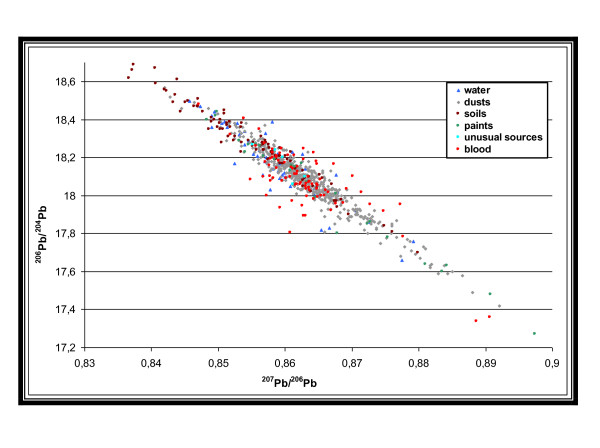
**^206^Pb/^204^Pb Vs ^207^Pb/^206^Pb of lead in blood and environmental samples. France, 2008-2009**.

For each type of source (dust from homes and communal areas are combined, as well as outdoor soil and wiped ground, because their isotopic signatures were very close), Figure 4 shows the distribution of the most discriminating LIRs, namely ^207^Pb/^206^Pb and ^206^Pb/^204^Pb. An analysis of variance (Mann-Whitney test) for these two LIRs showed a significant difference between the observed isotopic signatures of dust, ground and water and also between ground and paints. This statistical analysis of the whole collected samples was aiming to verify the possibility to assign a specific isotopic signature to each type of source for the final goal of identifying a child exposure source just using LIR from his blood. However, given the wide scatter of values, it was difficult to define a specific isotopic signature for each type of source due to the LIR overlap between types of sources. Therefore a child's exposure could not be determined by the simple comparison of its blood LIRs with typical and predefined LIRs of a type of source. This confirms the need for a specific assessment for each child requiring the collection of environmental samples from the residential environment of the child to compare with blood lead LIRs

### Identification of exposure sources

**Figure 5:**
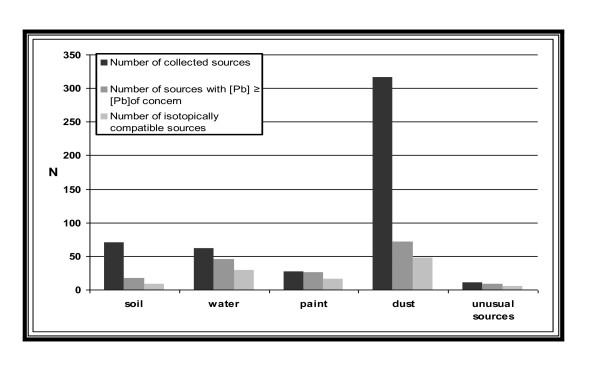
**Number of sources collected and sources eliminated by lead concentration and the six isotope ratios for children included in isotopic treatment. France, 2008-2009**. *Unusual sources include traditional dishes and cosmetics*

The number of samples collected ranged from four to twelve for each child (median = 7). Comparing the lead concentrations in the collected sources with the established concentrations of concern discarded 0 to nine sources (median = 4) per child. In addition, 0 to six (median = 1) of the remaining sources for each child (i.e. after lead concentration screening) were discarded using LIRs. Overall, the examination of lead concentrations and LIRs discarded 77% of the tested sources as illustrated by the Figure 5.

The enrolled children with B-Pb ≥ 25 μg/L and at least one potential source of exposure (after lead concentration screening) represent about 454,000 (CI_95% _= 305,000-604,000) French children aged six months-six years. Isotopic analyses were able to discriminate between potential sources for 75% of these children because the isotopic difference between sources was higher than the measurement error (DF > 1). It corresponds to 57% of children with B-Pb ≥ 25 μg/L, that are 7% of all French children (N = 331855, CI_95% _= 211,476-452,234).

The LIRs were able to eliminate at least one source of exposure for 53% (CI_95% _= 24-70%) of children for whom isotopic treatment was feasible. They identified, in addition to the concentrations measurements, a single suspected source of exposure for 56% (CI_95% _= 36-76%) of them. This corresponds to 41% (CI_95% _= 23-58%) and 39% (CI_95% _= 18-59%) respectively of children with B-Pb ≥ 25 μg/L with at least one potential source of exposure. Finally, the LIRs were useful for 30% (CI_95% _= 14-46%) of children with B-Pb ≥ 25 μg/L, and sufficient, in conjunction with the concentrations, for 32% (CI_95% _= 18-46%) of them. The concentrations (in environmental samples) alone were able to indicate a single source in 17.5% of children with B-Pb ≥ 25 μg/L.

No significant differences were observed when results were stratified according to the year of construction of the home or B-Pb. B-Pb were not significantly different (weighted t-test, p = 0.5) whether a single source was identified or not.

**Figure 6:**
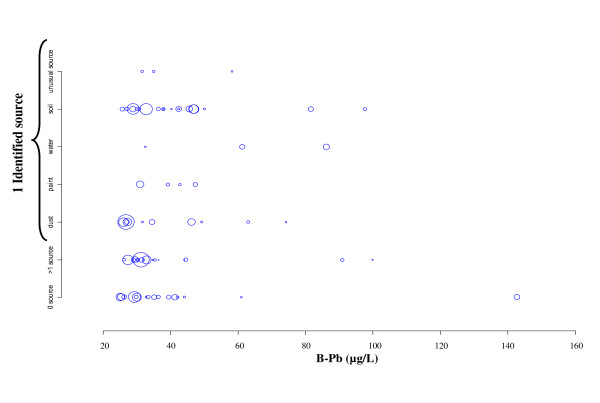
**Nature of sources identified by LIR against blood lead levels, France 2008-2009**. The radius of the bubble for each child is proportional to the sampling weight.

There was a variety of identified sources among children with a single source identified. The distribution of the type of identified unique source is given in Figure 6. Paints, dust, water, soil and unusual sources were pointed out as the exposure source in respectively 7% (CI_95% _= 0-14), 37% (CI_95% _= 11-64), 5% (CI_95% _= 0-11), 49% (CI_95% _= 22-77) and 1% (CI_95% _= 0-3) of children for whom a single source was identified.

When a single source of lead contamination was identified, there was a significant difference in children B-Pb when comparing identified types of sources (p = 0.04). The geometric mean of B-Pb was 30 μg/L in case of dust as a single contamination source, 36 μg/L for paint, 70 μg/L for water, 38 μg/L for soil, and 38 μg/L for unusual sources.

## Discussion

The use of isotopic analyses can provide additional information to ascribe lead exposure to a possible source. With the widespread use of quadrupole ICP-MS as a fast and relatively economical technique, it may be feasible to use isotopic analysis as a new tool in routine environmental investigations. This study assessed its usefulness for the first time at a nationwide level for identifying sources of lead exposure of children aged from six months to six years with moderate B-Pb.

The children included in the environmental investigation coordinated by CSTB were enrolled in the national study conducted by the InVS, whose representativeness is discussed by Etchevers et al. [14]. Four hundred and eighty four were included in the analysis. When comparing the group who declined participation to those who agreed, on the basis of access to free health insurance in France (CMU) - a very useful indicator because it is a risk marker for lead exposure in France [14]- there was no significant observed difference (p-value = 0.9).

ICP-MS was used instead of other techniques (Multicollector Mass Spectrometry) which are more accurate and precise. This choice was in line with the objective of the study, which was to evaluate the relevance of LIRs for practical analyses for public health action rather than for research purposes. Actually, quadrupole ICP-MS is more widely used in analysis laboratories. The discriminating factor provided a practical tool for evaluating the feasibility of LIRs and for identifying which LIRs to use to provide the greatest discrimination between lead sources in the residence and neighborhood. The population for whom LIRs may be relevant corresponded to 56% of children with B-Pb ≥ 25 μg/L, representing about 332,000 children (7% of French children aged from six months to six years). The method was useful and eliminated at least one unlikely source of exposure for 30% of children with B-Pb ≥ 25 μg/L, therefore avoiding unnecessary removal work. It identified a single suspected source of exposure for 32% of children with B-Pb ≥ 25 μg/L.

However, there are some limitations in the use of LIRs. Because environmental sampling cannot be exhaustive of exposure media, the main source(s) of exposure may not be identified, especially if lead exposure occurs outside the home or through diet. Moreover, a source may be wrongly considered to be isotopically compatible if it has the same isotopic composition as the real source or if its isotopic composition is between that of true sources. In addition, in cases of multi-source exposure, if the sources have different isotopic compositions, the blood will be located "partway" between these sources, which may be considered as incompatible. Furthermore, blood lead may be a combination of external lead and lead released from bones, in which case the isotopic signature of sampled sources may not agree owing to the presence of endogenous lead sources [16,31-33]. Finally, in this study, no diet samples were collected because dietary inputs consisted of many products for which the isotopic signature is a mix of several signatures from different lead sources. Also, no air samples were collected because of the very low concentrations of airborne lead in France, except possibly in the vicinity of some industrial plants. In addition to these inherent limitations of isotopic analyses, there are other limitations concerning this study. The B-Pb in children six months to six years have declined significantly in recent years and the number of children with B-Pb greater than 100 and 50 μg/L was much lower than expected. There were fewer children with relatively high blood lead levels in our sample than anticipated. As the lowest B-Pb may be due either to specific low exposures or to a random deviation from the baseline exposure (mainly food [34]), isotope analyses are less likely to identify the exposure source.

Despite a significant sampling campaign (more than 3,800 children sampled by the InVS and around 500 homes visited), there were only 125 children with B-Pb ≥ 25 μg/L in the sample, representing 590,175 children in the national population. Each child in our sample represented a large number (median: 2,040; mean: 4,796; range: 100-46,635) of children in the target population. Consequently, estimators are affected by large confidence intervals and any error (sampling, measurement, interpretation) for one child may significantly affect the estimates. In total, the population level estimates are interpreted as orders of magnitude, as shown by the confidence intervals.

The study evaluated the usefulness of LIRs for preventive actions. This led us to use leachable digestion method for the environmental samples (dust, paint, soil) which is prescribed for regulatory analysis in France. In order to compare to US studies, total digestion of the same samples was also performed using the method described by Le Bot et al. [26]. Comparison of results from leachable and total lead would be useful if French law were to change by prescribing total lead analyses. Lead isotope ratio uncertainties for total digestion can be obtained by combining uncertainties for both leachable and "pseudo total" lead (i.e. given the protocol of digestion, total lead from which half the leachable lead fraction was excluded), which leads to very large standard deviations, making them unusable for our study. For this reason, the LIRs used for comparison with results from leachable digestion were those obtained for "pseudo-total" lead. Comparison of results from leachable and "pseudo total" digestion included 58 children for whom it was possible, representing 269,019 children (CI_95% _= 169,288-368,750). Results for both types of digestion indicate that for 53% (CI_95% _= 31-76) of children both types of digestion show the usefulness of LIRs. For 34% (CI_95% _= 9-59) of children, LIRs were not useful for either digestion. Finally, for 13% (CI_95% _= 0-27) of children, LIRs were useful for one any type of digestion but not for the other. In all cases where both types of digestion led to a single source, the results were the same. The use of total and not "pseudo-total" lead should have led to minor differences because the fraction of unleachable lead is overrepresented in "pseudo-total" lead.

Lead concentrations are the primary means of detecting potential sources of overexposure. Sources with concentrations below predefined thresholds, based on reverse pharmacokinetic modeling, were eliminated. An uncertainty is associated with these concentrations of concern. Firstly, they were estimated from high amounts of ingested exposure media (the sensitivity of the test was preferred to its specificity), e.g. water consumption beyond normal. Secondly, these threshold concentrations of concern were applied to all children, without adjusting their B-Pb, water consumption, contact with the ground, unknown factors, etc. To test this potential influence, a sensitivity analysis was carried out using other concentrations of concern (twice the initial thresholds). As expected, as there were fewer potential sources, the usefulness of LIRs decreased from 53% to 42%: however, the order of magnitude remained broadly unchanged. In terms of identification of unique sources, a single source was identified for 41% of children instead of 56% with the initial threshold concentrations.

Another issue concerning the feasibility of isotopic analyses in routine environmental investigations is the choice of LIRs. As lead isotopes are strongly correlated, the LIRs yielding the greatest discrimination between sources are usually used. This study used the most discriminating LIRs for the French context, namely ^207^Pb/^206^Pb and ^206^Pb/^204^Pb. However, it is well known that using all lead isotopes could maximize discrimination of sources [16]. The measurement of the abundance of ^204^Pb lead, which is the most difficult to quantify owing to its much lower abundance, is sometimes omitted. A sensitivity analysis showed that results (in terms of identified sources) were different for 17.5% (CI_95% _= 1-34) of children. This proportion did not vary significantly according to B-Pb (p-value = 0.8). While some LIRs are less discriminating than others, this does not mean they are useless. The results in terms of sources identified were compared using six and then three and two LIRs successively for each child. The same results were obtained in almost all cases. For example, when interpreting only two ^207^Pb/^206^Pb and ^206^Pb/^204^Pb ratios, the same results were obtained as when six LIRs were used for 94% (CI_95% _= 78-100) of children. When ^208^Pb/^204^Pb was added to ^207^Pb/^206^Pb and ^206^Pb/^204^Pb, the results agreed with the use of six LIR for 98% (CI_95% _= 95-100) of children.

Overall, the identification of a single source of lead overexposure depended mainly on the following factors: (i) failure to sampling a source, (ii) the concentrations of concern and (iii) the uncertainty related to the sampling of the children. Moreover, the child's history, personal practices and details of his/her habits (including diet) were not considered. This would not be the case in an individual study for a public health campaign. It is noteworthy that isotope analyses were shown to be more useful in a targeted area in France with the highest B-Pb and more exhaustive environmental sampling [15].

## Conclusions

Current routine (i.e. relatively easy and not expensive to implement) identification of sources of lead exposure is based on the observation of child behavior and determination of the lead concentration in environmental samples. Use of LIRs has been suggested to reveal sources of exposure for children. To our knowledge, this study is, the first to assess the usefulness of LIRs to trace sources of lead, at a nationwide level. It applies to moderate B-Pb, especially important given the significant decrease in lead exposure in recent decades and the growing evidence of adverse effects at lower doses. The results, which must be interpreted with respect to the French context, show that LIRs have a valuable contribution for 30% of French children with B-Pb ≥ 25 μg/L. Despite the fact that these LIRs analyses are subject to stringent use conditions, especially concerning measurement accuracy, it appears that, when feasible, LIRs could throw new light on the environmental media to be incriminated. They can be used as additional tool in routine investigations to help environmental health officers to discard unlikely sources of exposure, thereby avoiding unnecessary remediation, which could result in further contamination. LIR's can be, and have been, applied in specific locations where there are appropriate situations where there are distinct isotopic signatures, such as in cases of contamination in mining areas.

These results will be completed by statistical analyses between blood lead levels, individual characteristics and indoor exposure to reveal determinants of moderate B-Pb. Combined results could be used to analyze action levels and intervention procedures to further reduce environmental lead poisoning.

## List of abbreviations

ICP-MS: Inductiely Coupled Plasma - Mass Spectrometry; B-Pb: Blood Lead Level; LIR: Lead Isotope Ratios; BC: Before the Christ Era; ANSES: Agence Nationale de Sécurité Sanitaire de l'Alimentation, de l'Environnement et du Travail (French Agency for Food, Environmental and Occupational Health and Safety); EFSA: European Food Safety Authority; IQ: Intellectual Quotient; InVS: Institut de Veille Sanitaire (French Institute for Public Health Surveillance); CSTB: Centre Scientifique et Technique du Bâtiment (Scientific and Technical Building Centre); CNIL: Commission nationale de l'informatique et des libertés (French Freedom of Information Commission); US-HUD: US Department of Housing and Urban Development; XRF: × Ray Fluorescence; PBPK: Physiologically Based Pharmacokinetic Model; IEUBK: Integrated Exposure Uptake Biokinetic Model for Lead; DF: Discriminating Factor; CI: Confidence Interval; CMU: Couverture Maladie Universelle (Free Health Insurance)

## Competing interests

The authors declare that they have no competing interests.

## Authors' contributions

YO conducted statistical analyses and interpretation and drafted the manuscript. BLB contributed to the study design, supervised isotopic analyses, helped in data interpretation and revised the manuscript. JP contributed to the study design, performed blood measurements. JPL performed data management, helped in statistical analyses and revised manuscript. CM supervised data acquisition and management and revised manuscript. AE supervised blood lead level study and recruitment of children, calculated sampling weights and revised the manuscript. DZN helped drafting and revised the manuscript. PG supervised the isotopic study, contributed to the study design, helped in data interpretation and drafting the manuscript. All authors read and approved the final manuscript.

## Supplementary Material

Additional file 1 **Intercalibration results**. This file contains results for the intercalibration between The LERES laboratory where environmental samples were analyzed and Lariboisière laboratory were blood samples were analyzed. Results were displayed for the six isotope ratios of lead. Click here for file
